# Associations Between Eczema and Attention Deficit Hyperactivity Disorder Symptoms in Children

**DOI:** 10.3389/fped.2022.837741

**Published:** 2022-03-30

**Authors:** Evelyn Xiu Ling Loo, Delicia Shu Qin Ooi, Minyee Ong, Le Duc Huy Ta, Hui Xing Lau, Michelle Jia Yu Tay, Qai Ven Yap, Yiong Huak Chan, Elizabeth Huiwen Tham, Anne Eng Neo Goh, Hugo Van Bever, Oon Hoe Teoh, Johan Gunnar Eriksson, Yap Seng Chong, Peter Gluckman, Fabian Kok Peng Yap, Neerja Karnani, Jia Xu, Karen Mei Ling Tan, Kok Hian Tan, Bee Wah Lee, Michael Kramer, Lynette Pei-chi Shek, Michael J. Meaney, Birit F. P. Broekman

**Affiliations:** ^1^Singapore Institute for Clinical Sciences, Agency for Science, Technology and Research, Singapore, Singapore; ^2^Department of Paediatrics, Yong Loo Lin School of Medicine, National University of Singapore, Singapore, Singapore; ^3^Human Potential Translational Research Programme, Yong Loo Lin School of Medicine, National University of Singapore, Singapore, Singapore; ^4^Department of Biostatistics, Yong Loo Lin School of Medicine, National University of Singapore, Singapore, Singapore; ^5^Khoo Teck Puat-National University Children’s Medical Institute, National University Hospital, National University Health System, Singapore, Singapore; ^6^Allergy Service, Department of Paediatrics, KK Women’s and Children’s Hospital, Singapore, Singapore; ^7^Respiratory Service, Department of Paediatrics, KK Women’s and Children’s Hospital, Singapore, Singapore; ^8^Department of Obstetrics & Gynaecology, Yong Loo Lin School of Medicine, National University of Singapore and National University Health System, Singapore, Singapore; ^9^Folkhälsan Research Center, Faculty of Medicine, University of Helsinki, Helsinki, Finland; ^10^Department of General Practice and Primary Health Care, University of Helsinki, Helsinki, Finland; ^11^Liggins Institute, University of Auckland, Auckland, New Zealand; ^12^Duke-NUS Medical School, Singapore, Singapore; ^13^Lee Kong Chian School of Medicine, Nanyang Technological University, Singapore, Singapore; ^14^Endocrinology Service, Department of Paediatrics, KK Women’s and Children’s Hospital, Singapore, Singapore; ^15^Department of Biochemistry, Yong Loo Lin School of Medicine, National University of Singapore, Singapore, Singapore; ^16^Bioinformatics Institute, Agency for Science, Technology and Research, Singapore, Singapore; ^17^Department of Maternal Fetal Medicine, KK Women’s and Children’s Hospital, Singapore, Singapore; ^18^Department of Pediatrics and of Epidemiology and Biostatistics, McGill University Faculty of Medicine, Montreal, QC, Canada; ^19^Sackler Program for Epigenetics & Psychobiology at McGill University, Montreal, QC, Canada; ^20^Ludmer Centre for Neuroinformatics and Mental Health, McGill University, Montreal, QC, Canada; ^21^Department of Psychiatry, McGill University, Montreal, QC, Canada; ^22^Department of Psychiatry, OLVG and Amsterdam UMC, VU University, Amsterdam, Netherlands

**Keywords:** atopy, attention deficit hyperactivity disorder, cytokines, eczema, gut microbiome dysbiosis

## Abstract

**Background:**

Epidemiological studies suggest a link between eczema and attention deficit hyperactivity disorder (ADHD), but underlying mechanisms have not been examined.

**Objective:**

We aim to investigate the association between eczema and subsequent ADHD symptoms in the Growing Up in Singapore Towards healthy Outcomes cohort and explore the role of pro-inflammatory cytokines and gut microbiome.

**Methods:**

The modified International Study of Asthma and Allergies in Childhood questionnaire and Computerized Diagnostic Interview Schedule for Children Version IV were administered to assess reported eczema within the first 18 months and presence of ADHD symptoms at 54 months, respectively. Skin prick testing at 18 months, cytokines in maternal blood during pregnancy and cord blood and the mediating role of the gut microbiome at 24 months were assessed.

**Results:**

After adjusting for confounders, eczema with or without a positive skin prick test was associated with doubling the risk of ADHD symptoms. No differences in maternal and cord blood cytokines were observed in children with and without eczema, or children with and without ADHD. Gut microbiome dysbiosis was observed in children with eczema and children with ADHD. Children with eczema also had lower gut bacterial Shannon diversity. However, the relationship between eczema and ADHD was not mediated by gut microbiome.

**Conclusion:**

Early life eczema diagnosis is associated with a higher risk of subsequent ADHD symptoms in children. We found no evidence for underlying inflammatory mechanism or mediation by gut microbiome dysbiosis. Further research should evaluate other mechanisms underlying the link between eczema and ADHD.

**Clinical Trial Registration:**

[https://clinicaltrials.gov/ct2/show/NCT01174875], identifier [NCT01174875].

## Introduction

The increasing incidence of eczema is paralleled by an increasing incidence of attention deficit hyperactivity disorder (ADHD) (1). Both eczema and ADHD are non-communicable diseases that carry a substantial economic burden and adversely affect quality of life (2, 3). Eczema is one of the earliest manifestations of allergic disease and affects approximately 20% of children; it generally manifests as dry and itchy skin on the face, elbow, and knee folds (4, 5). ADHD, characterized by inattention, hyperactivity, and impulsivity, is the most common mental health disorder in early childhood and occurs in 10% of children and adolescents (6).

Evidence from a number of studies suggests a link between eczema and ADHD. In a United States study including 354,416 children from 19 population-based surveys, eczema was associated with a 50% increased risk of developing ADHD compared to children without eczema (7). Similarly, in another United States study of 79,667 children aged 0–18 years, Yaghmaie and colleagues found that the odds of ADHD was two-fold higher in children with eczema than in those without eczema (5).

Despite the epidemiological associations between eczema and ADHD, studies have not evaluated the underlying mechanisms and common early-life factors that may mediate the association. Pro-inflammatory cytokines and the gut microbiome have been proposed to be involved in the development of both allergic and neurodevelopmental disorders (8). Eczema has been linked to increased production of Th2, Th17, and Th22 inflammatory cytokines (9). These pro-inflammatory cytokines can traverse the blood–brain barrier to activate the prefrontal cortex and increase susceptibility to cognitive disturbances (1). Inflammation may also be directly involved in the development of ADHD symptoms through microglial activation and tumor necrosis factor-α production, leading to altered central nervous system excitability (10). Also, alterations in gut microbiota composition have been reported in subjects with eczema (11) or ADHD (12).

Hence, the aim of our study is to investigate the association between eczema and subsequent ADHD symptoms in the Growing Up in Singapore Towards healthy Outcomes (GUSTO) cohort in a confirmatory analysis. We also explore associations of pro-inflammatory cytokines in maternal blood during pregnancy and cord blood with eczema and ADHD and the possible role of the gut microbiome in linking eczema and ADHD.

## Materials and Methods

### Study Design and Measurements

The GUSTO study is a prospective cohort study which recruited pregnant women attending their first-trimester antenatal dating ultrasound scan clinics at two major public maternity units in Singapore, KK Women’s and Children’s Hospital and National University Hospital from June 2009 to September 2010 (13). Pregnant women aged 18 years and above, from any one of the three major ethnic groups (Chinese, Malay, and Indian), Singapore citizen or permanent residents who had the intention of delivering in either hospital and staying in Singapore for at least the next 5 years, and who had agreed to donate their birth tissues were invited to participate. Women who had type 1 diabetes mellitus, or who were receiving chemotherapy or psychotropic drugs were excluded.

Trained interviewers gathered information on demographic characteristics, family history of allergy, socioeconomic data and lifestyle factors. Birth outcomes were measured and recorded after delivery. Ethical approval was obtained from the Domain Specific Review Board of Singapore National Healthcare Group (D/2009/021 approved on 26 February 2009) and the Centralised Institutional Review Board of SingHealth (2018/2767 approved on 2 March 2019). The conduct of this study was based on the guidelines in the Declaration of Helsinki. Written informed consent was obtained from the mothers after a detailed explanation of the study.

We used the modified International Study of Asthma and Allergies in Childhood (ISAAC) questionnaire at ages 3, 6, 9, 12, 15, and 18 months for evaluation of eczema, which was defined as maternally reported physician-diagnosed eczema based on the question: “Has your child ever been diagnosed with eczema?” at any timepoint, which included children with eczema ranging from mild to severe. The ISAAC questionnaire has been featured in several birth cohorts studying allergic outcomes (14, 15). We administered skin prick testing (SPT) at 18 months to assess hypersensitivity to the major relevant allergens in Singapore: cow’s milk, egg, peanut and house dust mites *Dermatophagoides pteronyssinus*, *Dermatophagoides farina* (Greer Laboratories, Lenoir, NC, United States), and *Blomia tropicalis* (developed in-house) (16). A positive SPT was defined as a positive SPT (wheal size ≥3 mm) to one or more of these allergens.

At 54 months, we administered the parent version of Computerized Diagnostic Interview Schedule for Children Version IV-Young Child (CDISC-YC), a highly structured diagnostic interview designed to be administered by lay interviewers to assess most of the commonly occurring mental health disorders in children (17). The mental health disorders in CDISC are based on the *Diagnostic and Statistical Manual of Mental Disorders-Fourth Edition Text Revised (DSM-IV)* (18). CDISC is one of the most extensively tested structural interviews. Although cultural differences in psychiatric symptoms cannot be ruled out, the CDISC has been used in different countries, and its performance, sensitivity and criterion validity have been verified in both clinical and community samples (17, 19–23). If symptoms of ADHD were present and sufficient to justify a possible diagnosis of ADHD according to DSM-IV, we classified this as “presence of ADHD.” This means that the ADHD symptoms must have persisted for at least 6 months to a degree that is inconsistent with developmental level and present in two or more settings (for example, at home and in school or other activities). However, the severity of these symptoms and the impaired social or academic functioning were not taken into account, given the young age (18). If symptoms were present but insufficient to meet the diagnostic criteria of ADHD, this was classified as “no ADHD.”

### Maternal and Cord Blood Cytokine Assays

Maternal blood samples were obtained between 26 and 28 weeks of pregnancy. Maternal and cord blood cytokines were assayed in plasma using customized Human ProcartaPlex Panels (Thermo Fisher Scientific, Massachusetts, United States), which uses Luminex xMAP technology in combination with DropArray bead plates (Curiox Biosystems, Singapore). Interferon gamma (IFNγ) and tumor necrosis factor-α (TNFα) were measured using single-molecule array assays on the SP-X platform (Quanterix Corp., United States). All values were corrected for plate-specific effects by centering at the median.

### Gut Microbiome Analysis

Stool samples were collected at 24 months. DNA extraction, 16S rRNA gene V4 region sequencing and data processing were performed. We followed the methods for gut microbiome analysis in our previous publications, with minor modification (24, 25). Briefly, DNA was extracted from 250 mg of stool samples based on manufacturer’s instructions (PowerSoil DNA isolation kit, catalog number 12855–100; Mo Bio). 16S rRNA gene V4 region amplification, library preparation and sequencing were performed using 250 base-pair paired-end reads on an Illumina MiSeq instrument at Argonne National Laboratories (University of Chicago). Raw sequencing data were processed using QIIME 1.9.0 and USEARCH v9.2.64 (26, 27). Both ends of the forward and reverse reads were truncated at the base where Q-value was no more than 2. The forward and reverse reads were merged into a complete read with the default setting in QIIME 1.9.0. The reads with length between 252 base pairs and 254 base pairs were retained. OTU was delineated at a similarity threshold of 97% using USEARCH v 9.2.64, and respective relative abundance and Shannon diversity index were obtained subsequently. All raw microbiome data are available at NCBI^1^ (Accession Number PRJNA668050).

### Statistical Analyses

Analyses were performed using the Statistical Package for the Social Sciences, Version 26 (IBM Cooperation, New York, NY, United States) and Stata version 16. Descriptive statistics for continuous variables are presented as mean (SD) when normality and homogeneity assumptions were satisfied, otherwise as median (IQR), and *n* (%) for categorical variables. Associations between maternally-reported diagnosis of eczema alone and in combination with a positive SPT, as well as presence of ADHD at 54 months as defined according to the CDISC structured interview, were assessed using Poisson regression, adjusting for demographic and other relevant covariates including mother history of allergy, maternal age, parity, child gender, ethnicity, childcare attendance in first year, exposure to tobacco smoke during pregnancy, gestational age, pre-pregnancy body mass index, child weight at birth, maternal education which have been shown to influence eczema and ADHD outcomes (28, 29). Differences in the levels of maternal and cord blood cytokines were assessed using linear regression with natural log transformation, adjusting for mother history of allergy, maternal age, parity, child gender, ethnicity, smoking status, pre-pregnancy body mass index (BMI) and maternal education. For gut microbiome analysis, the Mann–Whitney U test was performed to compare the bacterial Shannon diversity index. Linear regression was used to compare relative abundance of gut bacteria at 24 months between children with and without eczema within the first 18 months as well as abundance of gut bacteria at 24 months between children with and without ADHD at 54 months, with Bonferroni correction for multiple pairwise comparisons and adjustment for baseline values and three confounders (Fucosyltransferase 2, gender and duration of any breastfeeding that affects gut microbiome). Mediation analyses were performed using the Stata command Ldecomp to assess the role of the gut microbiome at 24 months in mediating the link between eczema in the first 18 months and ADHD at 54 months. We decomposed the total effect of eczema on ADHD into direct and indirect effects, adjusting for demographic and relevant covariates. Type 1 error rates for multiple outcomes of maternal and cord blood cytokines analysis and multiple mediators of mediation analysis were adjusted using the Benjamini-Hochberg procedure, with a false discovery rate at 0.20. Statistical significance was set at 2-sided *p* < 0.05.

## Results

### Study Population and Characteristics

A total of 288 mother–child pairs were included in the study after limiting the sample to children whose mothers completed the CDISC-YC at 54 months and the modified ISAAC questionnaire within the first 18 months. Of the 288 children, 158 (54.9%) were boys. Seventy-four (25.7%) children had at least once a reported diagnosis of eczema in the first 18 months. Seventy-two (25.0%) of the children had ADHD symptoms according to the CDISC at 54 months. The mothers’ median age at recruitment was 30.8 years (IQR 27.0–34.8). The majority of the mothers had 12 or fewer years of education [187 (65.6%)], were of Chinese ethnicity [142 (49.3%)], had no history of allergy [159 (56.8%)], and were not exposed to tobacco smoke during pregnancy [163 (60.4%)] (Table 1).

**TABLE 1:** Characteristics of the study population (*n* = 288).

	*n* (%) or median (IQR)
**Child sex**	
Female	130 (45.1%)
Male	158 (54.9%)
Maternal age (years)	30.8 (27.0 – 34.8)
**Maternal education level**	
≤12 years	187 (65.6%)
>12 years	98 (34.4%)
**Maternal ethnicity**	
Chinese	142 (49.3%)
Malay	99 (34.4%)
Indian	47 (16.3%)
Maternal history of allergy	121 (43.2%)
**Parity**	
Parous	159(55.2%)
Nulliparous	129(44.8%)
Childcare attendance in first year	18(6.5%)
**Exposure to tobacco smoke during pregnancy**	
Yes	107(39.6%)
No	163(60.4%)
Gestational age (years)	38.9 (37.9–39.9)
Pre-pregnancy BMI (kg/m^2^)	22.1 (19.7–25.5)
Child weight at birth (kg)	3.1 (2.9–3.4)
Eczema by 18M	74 (25.7%)
ADHD at 54 months	72 (25.0%)
ADHD at 54 months + Eczema by 18M	27 (9.4%)

*ADHD, attention deficit hyperactivity disorder; BMI: body mass index. Column values may not always add up to total due to missing values.*

### Maternal and Cord Blood Cytokine Assays

No significant differences were observed in the maternal blood cytokines C-reactive protein (CRP), IFNγ and TNFα between children with and without eczema or between children with and without ADHD (Table 2). Nor did we find significant differences in these same cytokines measured in cord blood from children with and without eczema or those with and without ADHD (Table 3).

**TABLE 2:** Differences in maternal blood cytokines.

		Unadjusted		Adjusted		
	*N*	B (95% CI)	*p*-value	*n*	B (95% CI)	*p*-value

Reference group: Subjects with no eczema						

IFNγ						
Eczema	42	0.19(-0.37 to 0.75)	0.507	37	0.14(−0.49 to 0.76)	0.669
No eczema	132			107		

**TNFα**						

Eczema	59	−0.01(−0.34 to 0.31)	0.929	49	−0.11(−0.45 to 0.23)	0.520
No eczema	178			147		

**CRP**						

Eczema	73	−0.25(−0.57 to 0.07)	0.125	61	0.03(−0.29 to 0.34)	0.870
No eczema	212			172		

**Reference group: Subjects with no ADHD**						

**IFNγ**						

ADHD	47	0.07(−0.47 to 0.61)	0.806	40	0.02(−0.60 to 0.64)	0.945
No ADHD	127			104		

**TNFα**						

ADHD	58	−0.23(−0.55 to 0.09)	0.164	46	−0.19(−0.54 to 0.16)	0.290
No ADHD	179			150		

**CRP**						

ADHD	71	0.05(−0.27 to 0.37)	0.766	56	0.11(−0.21 to 0.44)	0.489
No ADHD	214			177		

*ADHD, attention deficit hyperactivity disorder; CI, confidence interval; CRP, C-reactive protein; IFNγ, interferon gamma; TNFα, tumor necrosis factor α Benjamini–Hochberg correction with false discovery rate at 0.20 and n = 6 was applied.*

**TABLE 3:** Differences in cord blood cytokines.

		Unadjusted		Adjusted		
	*N*	B (95% CI)	*p*-value	*n*	B (95% CI)	*p*-value

Reference group: Subjects with no eczema						

IFNγ						
Eczema	30	0.07(−0.11 to 0.25)	0.454	25	0.11(−0.08 to 0.30)	0.257
No eczema	119			96		

**TNFα**						

Eczema	47	0.07(−0.01 to 0.15)	0.110	39	0.04(−0.04 to 0.13)	0.317
No eczema	163			133		

**CRP**						

Eczema	49	−0.03(−0.31 −to 0.25)	0.852	41	0.02(−0.27 to 0.30)	0.903
No eczema	174			143		

**Reference group: Subjects with no ADHD**						

**IFNγ**						

ADHD	31	−0.02(−0.21 to 0.16)	0.786	21	0(−0.2 to 0.2)	0.999
No ADHD	118			100		

**TNFα**						

ADHD	52	0.01(−0.07 to 0.09)	0.839	41	0.04(−0.05 to 0.13)	0.367
No ADHD	158			131		

**CRP**						

ADHD	52	0.11(−0.16 to 0.38)	0.428	41	−0.11(−0.40 to 0.18)	0.446
No ADHD	171			143		

*ADHD, attention deficit hyperactivity disorder; CI, confidence interval; CRP, C-reactive protein; IFNγ, interferon gamma; TNFα tumor necrosis factor α. Benjamini–Hochberg correction with false discovery rate at 0.20 and n = 6 was applied.*

### Associations Between Eczema and ADHD

A reported diagnosis of eczema within the first 18 months with and without a positive SPT was significantly associated with an increased risk of ADHD symptoms at 54 months [Adjusted relative risk (AdjRR) 2.5, 95%CI 1.1–6.0 and AdjRR 2.3, 95% CI 1.3–4.0, respectively] after adjusting for demographic and other covariates (Table 4).

**TABLE 4:** Poisson regression between eczema and ADHD at 54 months.

	Unadjusted		Adjusted	
	RR (95% CI)	*p*-value	RR (95% CI)	*p*-value
Eczema by 18 months	1.7 (1.1–2.8)	**0.024**	2.3(1.3–4.0)	**0.005^a^**
Eczema by 18 months + positive skin prick test	1.9 (0.9–4.0)	0.094	2.5(1.1–6.0)	**0.038^b^**

*ADHD, attention deficit hyperactivity disorder; CI, confidence interval; RR: relative risk. Significant p value in bold. In the adjusted model*,

*^a^81.9% of subjects (236 out of 288) and*

*^b^65.6% of subjects (189 out of 288) were used.*

### Gut Microbiome Dysbiosis at 24 Months

A lower gut bacterial Shannon diversity at 24 months was observed in 72 children with reported eczema within the first 18 months compared to the 250 without reported eczema (adj *p* < 0.05). No differences were observed between the 65 children with ADHD symptoms at 54 months and the 196 without such symptoms (adj *p* < 0.05) (Supplementary Figure 1).

**FIGURE 1:**
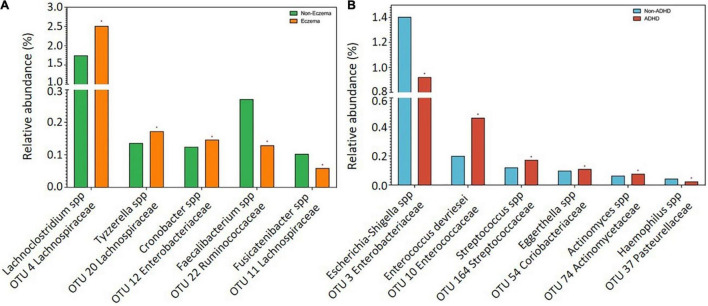
Comparisons of bacterial candidates in gut microbiome at 24 months between children with and without **(A)** eczema by 18 months (*n* = 72 vs 250), **(B)** ADHD symptoms at 54 months (*n* = 65 vs 196). Data are presented as median. Only significant bacteria (adjusted *p* < 0.05) with reference to controls were shown.

The dominant gut bacterial candidates (>0.1% relative abundance) were further analyzed and those which showed significant differences between groups were plotted in term of relative abundance, as shown in Figure 1. Increases in relative abundance of *Lachnoclostridium* spp. and *Tyzzerella* spp. (Lachnospiraceae family); and *Cronobacter* spp. (Enterobacteriaceae family) and decreases in *Faecalibacterium* spp. (Ruminococcaceae family) and *Fusicatenibacter spp*. (Lachnospiraceae family) were observed in children with reported eczema compared to those without (adj *p* < 0.05) (Figure 1A). The relative abundance of *Escherichia* or *Shigella* spp. (Enterobacteriaceae family) and *Haemophilus* spp. (Pasteurellaceae *family*) was decreased, while that of *Enterococcus devriesei* (Enterococcaceae family), *Streptococcus* spp. (Streptococcaceae family), *Eggerthella* spp. (Coriobacteriaceae family), and *Actinomyces* spp. (Actinomycetaceae family) was increased in children with ADHD symptoms at 54 months compared to those without (adj *p* < 0.05) (Figure 1B).

### Mediating Role of Gut Microbiota in the Association Between Eczema and Subsequent ADHD

No mediation effect (overall indirect effect) was observed for any gut microbiota in the association between eczema reported within the first 18 months and ADHD symptoms at 54 months (Table 5).

**TABLE 5:** Mediation effect of gut microbiome on ADHD at 54 months (*n* = 129).

Mediators at month 18	Indirect effect	Adjusted *p*-value	% mediated
OTU3	0.02(−0.10 to 0.14)	0.732	1.9%
OTU4	0.02(−0.09 to 0.13)	0.705	1.9%
OTU5	0.01(−0.21 to 0.22)	0.947	0.7%
OTU10	0.02(−0.15 to 0.19)	0.834	1.6%
OTU11	0.04(−0.13 to 0.22)	0.621	4.1%
OTU12	−0.01(−0.14 to 0.11)	0.823	NA
OTU20	−0.06(−0.26 to 0.13)	0.534	NA
OTU22	0.05(−0.08 to 0.18)	0.466	4.6%
OTU37	0.02(−0.20 to 0.23)	0.887	1.4%
OTU54	0.001(−0.107 to 0.109)	0.989	0.1%
OTU68	0.05(−0.31 to 0.40)	0.813	4.2%
OTU74	−0.03(−0.18 to 0.11)	0.666	NA
OTU164	0.02(−0.37 to 0.41)	0.904	2.1%
Overall	−0.01(−0.94 to 0.91)	0.938	NA

*ADHD, attention deficit hyperactivity disorder; NA, not applicable; OTU, operational taxonomic unit. Benjamini–Hochberg correction with false discovery rate at 0.20 and n = 13 was applied. NA:% mediated is not computed due to inconsistent mediation.*

## Discussion

In the prospective GUSTO cohort, we observed that maternally reported diagnosis of eczema within the first 18 months of life with or without a positive SPT was associated with a doubling of the risk of ADHD symptoms. These findings are consistent with previous reports of this association (30, 31). While there are studies investigating the effects of cytokine levels and gut microbiome on eczema and ADHD separately (32, 33), to our knowledge, this is the first study to examine the roles of cytokines and gut microbiome in mediating the association between eczema and ADHD.

A cross-sectional study by Schmitt et al. found that children with eczema were approximately 1.5 times more likely to have ADHD (30). Another prospective birth cohort study in Germany reported that eczema by 4 years was associated with ADHD diagnosis by 8 years (31). Conversely, other studies did not report significant associations between eczema and ADHD (34, 35). These include the BAMSE birth cohort, which reported that preschool eczema was not associated with use of ADHD medication at school age (34). One possible reason for the difference is that some children with ADHD symptoms did not receive medication. Suwan et al. reported no significant association between eczema and ADHD in a cross-sectional case-control study of children aged 5–15 years (35). However, the design of the latter study was unable to assess an association with eczema at younger ages.

Eczema and ADHD are linked to the release of pro-inflammatory cytokines (8). In our study, however, we observed no differences in maternal or cord blood cytokines between children with and without eczema, nor between children with and without ADHD symptoms. Our findings are contrary to results reported by other studies (36, 37). A possible reason for the different results may be that we assayed cord blood without stimulation and cord blood levels of IFNγ without stimulation are generally low (38). Supportive evidence is provided by an Indonesian birth cohort study that also found no association between unstimulated cord blood IFNγ levels and eczema development by six months (39). Similarly, the Hokkaido Study on Environment and Children’s Health reported no associations between unstimulated cord blood TNFα levels and hyperactivity or inattention issues in children (40). Another reason for the absence of differences in maternal or cord cytokines between children with and without eczema, nor between children with and without ADHD symptoms that we observed may be due to limited immune profiling. While the Newborn Epigenetics Study and LINA cohort reported associations between maternal IL-12p70, IL-17A, IL-1β, and IL-13 levels and child neurodevelopment (36, 37), we did not measure these cytokines. We also assayed cytokines from maternal blood during pregnancy and cord blood which is not indicative of subsequent inflammation that may be brought about by gut microbiome dysbiosis that we have observed in children with eczema as well as in children with ADHD.

Gut microbiome dysbiosis may be linked to allergic and neurodevelopmental diseases. We observed that relative abundance of *Lachnoclostridium* spp. was substantially higher in the gut microbiome of subjects with eczema in comparison to subjects without eczema. The pro-inflammatory role of *Lachnoclostridium* had been shown in murine models in which gut *Lachnoclostridium* abundance was higher in wild-type eczema mouse models as compared to anti-inflammatory interleukin-37b knock-in mice (41). Gut *Lachnoclostridium* is also found in higher abundance in patients with inflammatory Crohn’s disease (42). We propose that higher relative abundances of *Lachnoclostridium* may be linked to inflammation and result in eczema. We also observed lower relative abundance of *Faecalibacterium spp.* and *Fusicatenibacter* spp. in the gut microbiome of children with eczema. Lower abundance of *Fusicatenibacter* in the gut is linked to higher levels of inflammatory fecal calprotectin (43). High fecal calprotectin levels in offspring at 2 months of age has been associated with eczema development by age 6 years (44). *Faecalibacterium* and *Fusicatenibacter* are also short-chain fatty acid (SCFA) producers which serve as an important energy source and exhibit anti-inflammatory properties in the host (45).

In children with ADHD symptoms compared to children without ADHD symptoms, we observed a substantial reduction in relative abundance of gut *Escherichia-Shigella* spp. and increase in gut *Enterococcus devriesei*. Supportive evidence is provided by murine models where mice colonized with gut microbiota from ADHD patients had lower concentrations of gut *Escherichia* or *Shigella* spp. as compared to controls (46). *Escherichia* is also a SCFA producer that can modulate neurotransmitters and neurotrophic factors, such as brain-derived neurotrophic factor, which is important for neurogenesis and in turn may influence the development of ADHD (45). A study of Chinese schoolchildren reported higher *Enterococcus* abundance in children with ADHD (47).

We found no indications that the gut microbiome mediates the association we observed between eczema and later ADHD symptoms. Further research would help to understand the role of the gut microbiome in these diseases.

Strengths of our study include the frequent, prospective follow-up of subjects using validated questionnaires and a structured clinical interview to assess presence of ADHD symptoms. Limitations of our study include maternally reported diagnosis of eczema, although we objectively assessed atopy using SPT in a subsample. Also, we have explored the relation with a “presence of ADHD” based on the number of ADHD symptoms present, but without taking into account the degree of impairment in functioning. Also, the symptoms of ADHD were assessed using parental reports without clinical observation of the children. The cytokines were only evaluated in maternal blood during pregnancy and in cord blood and not in later timepoints. Finally, we did not study other factors and possible mechanisms, such as maternal mental health state, sleep disturbances, epigenetics, gene expression and mitochondrial dysfunction, that may underlie the pathogenesis of allergic and neurodevelopmental disorders (8). For instance, a study of Italian children with autism spectrum disorder by Lamanna et al. reported that presence of allergies and family history of psychosis accounted for 25% of variance in ADHD severity, hence highlighting the importance of examining comorbidities in understanding the mechanisms linking eczema and ADHD development (48).

In conclusion, we observed that early life eczema is associated with a higher risk of later presence of ADHD symptoms in children. We found no indications for an association with maternal blood or cord cytokines. Gut microbiome dysbiosis was a common feature of both eczema and ADHD, but association between eczema and ADHD was not mediated by the gut microbiome. More studies are clearly warranted to elucidate the link between eczema and ADHD, as well as the biological mechanisms underlying the link.

## Data Availability Statement

The data that support the findings of this study are available on request from the corresponding author. The data are not publicly available due to privacy or ethical restrictions. All raw microbiome data are available at NCBI (https://www.ncbi.nlm.nih.gov) (accession number PRJNA668050).

## Ethics Statement

Ethical approval was obtained from the Domain Specific Review Board of Singapore National Healthcare Group (D/2009/021 approved on 26 February 2009) and the Centralised Institutional Review Board of SingHealth (2018/2767 approved on 2 March 2019). The conduct of this study was based on the guidelines in the Declaration of Helsinki. Written informed consent was obtained from the mothers after a detailed explanation of the study. Written informed consent to participate in this study was provided by the participants’ legal guardian/next of kin.

## Author Contributions

EL conceptualized and designed the study, designed the methodology, performed data analysis, drafted the initial manuscript as well as reviewed and revised the manuscript. DO, MO, HL, and MT drafted the initial manuscript as well as reviewed and revised the manuscript. LT performed the data analysis, drafted the initial manuscript as well as reviewed and revised the manuscript. QY designed the methodology, performed data analysis, drafted the initial manuscript as well as reviewed and revised the manuscript. YHC designed the methodology, performed data analysis as well as reviewed and revised the manuscript. ET, AG, HV, OT, JE, YSC, PG, FY, KMT, KHT, NK, BL, LS, and MM conceptualized and designed the study as well as reviewed and revised the manuscript. JX performed the data analysis as well as reviewed and revised the manuscript. MK designed the methodology, drafted the initial manuscript as well as reviewed and revised the manuscript. BB conceptualized and designed the study, supervised the project as well as reviewed and revised the manuscript. All authors agree to be accountable for the work.

## Conflict of Interest

YSC and NK are part of an academic consortium that has received research funding from Abbot Nutrition, Nestle and Danone. The remaining authors declare that the research was conducted in the absence of any commercial or financial relationships that could be construed as a potential conflict of interest.

## Publisher’s Note

All claims expressed in this article are solely those of the authors and do not necessarily represent those of their affiliated organizations, or those of the publisher, the editors and the reviewers. Any product that may be evaluated in this article, or claim that may be made by its manufacturer, is not guaranteed or endorsed by the publisher.

## Footnotes

1
https://www.ncbi.nlm.nih.gov

## Abbreviations

AdjRR: adjusted RR
ADHD: attention deficit hyperactivity disorder
BMI: body mass index
CDISC-YC: Computerized Diagnostic Interview Schedule for Children Version IV Young Child
CRP: C-reactive protein
DSM-IV: Diagnostic and Statistical Manual of Mental Disorders-Fourth Edition Text Revised
GUSTO: Growing Up in Singapore Towards healthy Outcomes
IFN γ: interferon gamma
SCFA: short-chain fatty acid
SPT: skin prick testing
TNF α: tumor necrosis factor α.
