# Supplementary material for: Associations Between Eczema and Attention Deficit Hyperactivity Disorder Symptoms in Children
Source: Front Pediatr. 2022 Mar 30;10:837741. doi: 10.3389/fped.2022.837741 (PMC9007142; doi:10.3389/fped.2022.837741)
Supplement: Supplementary file 1 [file Image_1.pdf]

Supplementary Figure

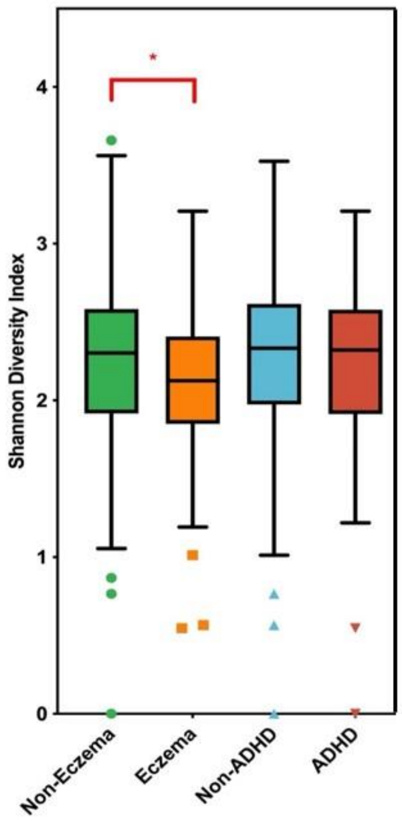

Supplementary Figure 1. Comparisons of gut microbial diversity assessed by Shannon diversity index at 24 months between children with eczema and without and children with ADHD and without subjected to Bonferroni correction. Maximum, minimum, 25th percentile, 75th percentile, and median were used for box and whisker plots.

* indicates significance at adj $p<0.05$
